# Interventions to reduce occupational burnout in general practitioners: a systematic review and meta-analysis protocol

**DOI:** 10.3389/fpubh.2026.1748369

**Published:** 2026-03-31

**Authors:** Wei Xi, Zhonghan Li, Shanping Chen, Peisheng Xia, Zihao Zhang, Yanting Chen, Junxian Li, Xiaolong Shuai, Chuan Zou

**Affiliations:** 1 School of Medical and Life Sciences, Chengdu University of Traditional Chinese Medicine, Chengdu, China; 2 Hospital of Chengdu University of Traditional Chinese Medicine, Chengdu, China; 3 The Department of Geriatric Medicine, Affiliated Fifth People’s Hospital of Chengdu University of Traditional Chinese Medicine, Chengdu, China; 4 Health Management Center, The First People’s Hospital of Guangyuan, Guangyuan, China; 5 General Practice Ward/International Medical Center Ward, General Practice Medical Center, West China Hospital, Sichuan University, Chengdu, China; 6 Department of General Practice, Affiliated Fifth People’s Hospital of Chengdu University of Traditional Chinese Medicine, Chengdu, China

**Keywords:** burnout, general practitioners, intervention, meta-analysis, occupational stress, primary health care, systematic review

## Abstract

**Background:**

General practitioners (GPs) are at high risk of burnout, which threatens physicians’ mental health, care quality, and the sustainability of primary health care systems. Although a growing number of interventions have been developed to prevent or reduce burnout among GPs, their effectiveness and the mechanisms through which they work remain unclear.

**Methods:**

We will conduct a systematic review and meta-analysis of interventions aimed at preventing or reducing burnout in GPs, following the Cochrane Handbook and the PRISMA 2020 statement. Randomized controlled trials, cluster-randomized trials, and non-randomized controlled studies evaluating individual-, group-, organisational-, or system-level interventions will be eligible. We will search PubMed (MEDLINE), Embase (Elsevier), Web of Science Core Collection, CNKI, Wanfang Data, and VIP (Weipu) and other databases from inception, without language restrictions. Two reviewers will independently screen studies, extract data and assess risk of bias using the revised Cochrane risk-of-bias tool (RoB 2) and the ROBINS-I tool. Where appropriate, we will pool effect sizes for overall burnout and its dimensions using random-effects models, and explore heterogeneity and subgroup effects based on the job demands–resources model. When meta-analysis is not feasible, we will provide a narrative synthesis. Certainty of the evidence will be assessed with GRADE.

**Systematic review registration:**

PROSPERO, Identifier CRD420251064119.

## 1 Introduction

Burnout is defined in the WHO’s International Classification of Diseases, 11th Revision (ICD-11) as a syndrome resulting from chronic workplace stress that has not been successfully managed (1). Maslach et al. (2, 3) conceptualise three core dimensions: ① emotional exhaustion; ② depersonalisation—or a cynical, negative attitude towards work; and ③ reduced professional efficacy.

The prevalence of physician burnout has remained persistently high and poses a serious challenge to health systems worldwide (4, 5). A systematic review covering 22,177 general practitioners (GPs) across 29 countries estimated moderate to high levels of burnout among GPs globally (6). Burnout compromises clinicians’ physical and mental health and has serious clinical and system-level consequences (7, 8). Compared with their non-burned-out peers, physicians with burnout are nearly twice as likely to report patient-safety incidents and demonstrate significantly poorer professional performance and lower patient satisfaction (9). Over time, burnout erodes job satisfaction and markedly increases turnover intention and workforce attrition, undermining the quality and sustainability of care (10, 11).

A substantial body of evidence indicates that GPs are among the specialties reporting the highest rates of burnout (12–14). As gatekeepers in primary care, GPs shoulder broad and complex clinical responsibilities, facing a constellation of specialty-specific risk factors and stressors that differ from other disciplines (15–17). First, the nature of general practice requires coping with heavy workloads and undifferentiated clinical presentations: within limited consultation time GPs manage large patient volumes whose conditions are diverse, often unscreened, and clinically complex, demanding breadth of knowledge, efficient decision-making and effective stress management (12, 15, 18). Second, many GPs work in resource-constrained settings with inadequate diagnostic equipment, limited referral pathways and insufficient social support services—an imbalance between job demands and available resources that is pivotal in precipitating burnout (6, 19, 20). Third, escalating administrative burdens—particularly electronic health record (EHR)-related clerical work—have been shown to be major drivers of physician burnout, consuming time that would otherwise be devoted to clinical care (21). Finally, unlike hospital-based multidisciplinary environments, GPs may experience occupational isolation with limited timely peer support and professional exchange (22).

Although reviews of burnout interventions among physicians exist, there remains a lack of GP-specific evidence syntheses that systematically compare intervention types and provide theory-driven analyses. Given the distinctive practice context of GPs, effectiveness findings from other specialties may not be directly generalisable. Therefore, a dedicated systematic review and meta-analysis of interventions targeting GP burnout is warranted.

## 2 Objectives

This review aims to systematically identify, appraise and synthesise all available evidence on interventions designed to reduce occupational burnout among GPs. The primary objective is to estimate the overall effectiveness of these interventions in reducing GP burnout. The secondary objective is to conduct a theory-driven classification of interventions and, through prespecified subgroup analyses, compare the relative effectiveness of different categories and explore potential factors underlying between-study differences. The findings will inform strategies to support the GP workforce, improve the quality of primary care, and guide the design of future studies.

Specifically, we seek to answer the following questions:

① What is the overall effectiveness of interventions intended to reduce GP burnout?

② How does effectiveness differ across intervention categories, and which intervention functions or theoretical pathways are most closely associated with reductions in burnout?

③ What factors potentially account for differences in intervention effects?

## 3 Methods and analysis

This study is a prospectively registered systematic review and meta-analysis protocol. It was registered with PROSPERO on 1 June 2025 (NO. CRD420251064119). The protocol has been prepared in accordance with PRISMA-P (23); the final report will follow PRISMA 2020 (24), and the methods are based on the Cochrane Handbook for Systematic Reviews of Interventions (25).

### 3.1 Eligibility criteria

Studies will be included only if they meet both the study-level and reporting-level criteria below.

#### 3.1.1 Study characteristics

① Design: randomised controlled trials (RCTs), cluster-randomised trials (cRCTs), and non-randomised comparative studies with a control group. ② Participants: general practitioners (GPs) practising in primary care settings. Mixed-sample studies will be eligible only if GPs constitute ≥70% of the sample or GP-specific data are separately extractable. The ≥70% threshold was chosen to balance target population representativeness with the ecological validity of practice-level interventions in real-world primary care settings, where minor multidisciplinary overlap commonly exists. To account for potential residual contamination from other specialties, we will conduct a pre-specified sensitivity analysis excluding these mixed-sample studies to rigorously confirm the robustness of the GP-specific findings. Residents/trainees or medical students will be included only when GP subgroup data are reported separately. ③ Interventions: single- or multicomponent interventions with an explicit aim to reduce occupational burnout or its core dimensions. ④ Comparators: any comparator (e.g., usual care, wait-list, active control). ⑤ Outcomes: burnout measured using validated instruments with established reliability, including the Maslach Burnout Inventory (MBI) and its variants, the Oldenburg Burnout Inventory (OLBI), the Copenhagen Burnout Inventory (CBI), or other validated tools (2, 26, 27).

#### 3.1.2 Reporting characteristics

① Timeframe and language: no restrictions. ② Publication status: both published and unpublished studies are eligible. ③ Data availability: studies must provide the minimum data required to compute effect sizes; if, after contacting study authors, the necessary data remain unavailable, the study will be excluded.

### 3.2 Information sources and search strategy

We will search PubMed (MEDLINE), Embase (Elsevier), Web of Science Core Collection, CNKI, Wanfang Data, and VIP (Weipu). Grey literature will be searched via Google Scholar and Baidu. Furthermore, we will search clinical trial registries, specifically ClinicalTrials.gov and the WHO International Clinical Trials Registry Platform (ICTRP). We will also handsearch the reference lists of included studies and relevant reviews identified during study selection, and consult domain experts to locate additional records not captured by database searches.

The search strategy will be structured around three core concepts—general practitioners, occupational burnout, and interventions—and will combine controlled vocabularies (e.g., MeSH, Emtree) with free-text terms, tailored to each database’s syntax. The full search strategies for the six databases are provided in Appendix. No search limits will be applied.

### 3.3 Study selection

Search results will be imported into EndNote 21 (Clarivate) for management. The software’s built-in automated duplicate detection will be used for an initial pass to identify and remove obvious duplicates. Thereafter, two reviewers will manually verify the remaining library—sorting by title, author and year—to identify and remove any residual duplicates that may have been missed by the automated tool. After de-duplication, records and screening data will be managed in Covidence (Veritas Health Innovation). Before formal screening, we will conduct a pilot calibration exercise in which two reviewers independently screen 30 titles/abstracts. Inter-rater agreement will be assessed using Cohen’s *κ*; if *κ* < 0.80, the inclusion/exclusion criteria and decision rules will be clarified and refined. For the main screening, two reviewers will independently screen titles and abstracts, recording a reason for exclusion for each record. Articles judged potentially eligible will then undergo full-text assessment against the eligibility criteria. Disagreements at any stage will be resolved by discussion and, if needed, adjudication by a third reviewer, with decisions documented. Covidence will automatically generate a PRISMA 2020 flow diagram to summarise the study selection process.

### 3.4 Data extraction and data items

Following study selection, two reviewers will independently extract data in duplicate using a standardised extraction form and cross-check each other’s entries for completeness and accuracy. Before formal extraction, the form will be pilot-tested on a small sample to ensure consistent interpretation of all items. Any discrepancies between extractors will be resolved through discussion; if consensus cannot be reached, a third reviewer will adjudicate. The calibration procedures and all decisions will be documented in detail. The data items are listed in Table 1. For missing or unclear information, we will contact the original study authors to obtain additional details; all correspondence and attempts will be recorded and archived. Specifically, the classification of interventions into JD-R categories (demand-reducing, resource-enhancing, or dual-pathway) will be performed independently by two reviewers in duplicate. For complex, multicomponent interventions, reviewers will code based on the primary active ingredients explicitly described by the study authors. Any disagreements in JD-R pathway classification will be resolved through consensus discussion, with a third senior reviewer acting as an arbiter if consensus cannot be reached.

**Table 1:** Data extraction items.

Category	Data extraction items
A Study and publication information	A1 Identification details: first author, year of publication, country where the study was conducted
	A2 Study design
	A3 Funding source(s) and conflicts of interest
B Participant characteristics	B1 Practice setting/type of workplace
	B2 Sample size
	B3 Inclusion and exclusion criteria
	B4 Demographic characteristics: age, sex distribution, years in practice
C Intervention and comparator information	C1 Description of the intervention content
	C2 Intervention dose and intensity: total duration; total contact time; delivery frequency and session length; delivery mode; facilitator background; whether protected work time was provided
	C3 Description of the comparator/control
D Theoretical framework coding	D1 JD-R classification: demand-reducing; resource-enhancing; dual-pathway (combined) intervention
	D2 Rationale for the JD-R classification
E Outcomes and synthesis data	E1 Burnout measurement instrument
	E2 Reported subdimensions: emotional exhaustion (EE); depersonalisation (DP); personal accomplishment (PA)
	E3 Definition of “burnout”: any specified cut-off used to define “burnout” or “high burnout”
	E4 Continuous outcomes: number of participants (*N*), mean, standard deviation (SD), confidence interval (CI), standard error (SE), *p* value
	E5 Dichotomous outcomes: number of events and group totals
	E6 Follow-up time points
	E7 Adverse events

### 3.5 Risk of bias assessment

We will assess risk of bias for each included study using Cochrane-recommended methodological tools. Randomised controlled trials will be appraised with the Cochrane Risk of Bias 2 (RoB 2) tool (28); for cluster-randomised trials, we will use the RoB 2—Cluster version (29). Non-randomised comparative studies will be evaluated using ROBINS-I (Risk Of Bias In Non-randomized Studies of Interventions) (30). Two reviewers will independently apply the relevant tool to each study, recording domain-level judgements and justifications on standardised forms. Results will then be compared and discrepancies resolved through discussion; if consensus cannot be reached, a third reviewer will adjudicate.

Risk-of-bias findings will inform the narrative synthesis, sensitivity and subgroup analyses, heterogeneity exploration, and the certainty-of-evidence assessment (GRADE) (31). Visualisations will be produced using robvis (traffic-light plots and weighted bar charts).

### 3.6 Analysis, data synthesis, publication bias and reporting

For studies deemed comparable, we will conduct a quantitative meta-analysis. For continuous outcomes, the effect measure will be the standardised mean difference (SMD) with 95% confidence intervals (CIs), corrected using Hedges’ g. To enhance the practical interpretation of the pooled SMDs, and in the absence of universally established Minimal Clinically Important Differences (MCIDs) for burnout among GPs, we will interpret effect sizes using Cohen’s commonly accepted benchmarks: ~0.2 as a small effect, ~0.5 as a moderate effect, and ~0.8 or greater as a large effect. For dichotomous outcomes, the primary effect measure will be the risk ratio (RR) with 95% CIs; where the available data permit only calculation of an odds ratio (OR), we will report the OR as an alternative and explicitly note this choice. Missing standard deviations (SDs) will be derived from SEs, CIs, *t* values or *p* values using the formulae provided in the Cochrane Handbook for Systematic Reviews of Interventions (25). Given the complexity of interventions and contextual variability, meta-analyses will use a random-effects model, estimating the between-study variance *τ*^2^ via restricted maximum likelihood (REML) and applying the Hartung–Knapp–Sidik–Jonkman (HKSJ) adjustment for CIs. Statistical heterogeneity will be assessed using *I*^2^; values of ≈25% will be interpreted as low, >50% as moderate, and >75% as high heterogeneity (25).

Prespecified subgroup analyses will examine: intervention categories based on the Job Demands-Resources (JD-R) framework, level of target (organisational vs. individual), study design, risk of bias, and participant seniority. We will initially perform separate meta-analyses for randomized trials and non-randomized studies. Where outcomes and effect measures are harmonised and broadly aligned in both direction and magnitude, we will examine heterogeneity between randomised and non-randomised evidence. We will consider the results sufficiently robust to pool across study designs only if the test for subgroup differences yields a *p*-value >0.10, and if the non-randomized studies are judged to be at low or moderate risk of bias according to the ROBINS-I tool. If these criteria are not met, the results will remain stratified by study design.

For special study designs, cluster-randomised trials will be adjusted for clustering using the design effect; when the intracluster correlation coefficient (ICC) is unreported, we will preferentially borrow plausible ICCs from the same domain or conduct sensitivity analyses across plausible ICC values to evaluate their influence on pooled results. For multi-arm trials, intervention arms sharing similar core mechanisms will be combined before comparison with the control; when arms differ materially, we will select the arm most pertinent to the review question for the primary comparison. All decisions and their rationales will be documented and reported.

If a meta-analysis includes ≥10 studies, we will assess publication bias/small-study effects using the Egger test and funnel plots (32). Where quantitative synthesis is inappropriate, we will undertake a structured narrative synthesis following the SWiM guideline (33). Regarding adverse events or unintended consequences of the interventions (e.g., paradoxical increases in time pressure or workload resulting from program participation), we anticipate high reporting heterogeneity. Therefore, these data will be synthesized narratively following the SWiM reporting guideline and presented in a dedicated summary table. This qualitative synthesis will be directly integrated into our final assessment of the interventions’ overall benefit-to-harm profile. The certainty of evidence for all primary outcomes will be rated using GRADE, and key findings will be summarised in a Summary of Findings (SoF) table.

## 4 Discussion

### 4.1 Innovations of this study

The Job Demands-Resources (JD-R) model is one of the most widely validated theoretical frameworks in research on occupational burnout (34, 35). It categorises work environment factors into two broad classes: job demands and job resources. According to its canonical assumptions, demands and resources influence employees’ mental and physical states via two relatively distinct yet related pathways: the health-impairment pathway and the motivational pathway. Along the health-impairment pathway, excessive job demands deplete energy and psycho-physiological resources, leading to sustained stress and emotional exhaustion, which can develop into burnout. Along the motivational pathway, adequate job resources stimulate positive motivation and work engagement, satisfy psychological needs, and thereby enhance engagement while preventing cynicism and turnover intention. In short, increased demands tend to precipitate adverse outcomes such as burnout, whereas richer resources buffer or reduce burnout and foster positive engagement.

Within this framework, demand-reducing interventions are expected to attenuate or disrupt the health-impairment pathway by lowering upstream stressors; resource-enhancing interventions are expected to strengthen the motivational pathway by boosting motivation and adaptive capacity, thereby offsetting the impact of stress on burnout. Accordingly, measures intended to alleviate burnout can be interpreted within the JD-R model as either (a) reducing excessive workload and stressors to prevent energy depletion, or (b) providing more support, feedback and autonomy to enhance motivation and resilience, thereby reducing the occurrence of burnout.

This review introduces a JD-R-informed subgroup analysis, classifying interventions into three categories for comparative evaluation: ① demand-reducing interventions; ② resource-enhancing interventions; and ③ dual-pathway (combined) interventions that both reduce demands and increase resources. This approach allows us to test whether interventions aligned to different pathways exert systematically different effects on burnout outcomes, to provide clearer theory-based interpretability of findings, and to offer decision-relevant guidance to clinicians and policymakers on which specific intervention strategies are most likely to be effective (see Table 2).

**Table 2:** Categories of interventions based on the JD-R model and representative measures.

Intervention type (JD-R pathway orientation)	Representative examples of occupational burnout interventions
Demand-reducing interventions	Reduce workload; redistribute high-intensity or emotionally taxing tasks through job rotation. Schedule protected breaks and recovery time to prevent sustained overwork and fatigue, reducing excessive job stress at source.
Resource-enhancing interventions	Provide greater social support and increase job autonomy; deliver timely feedback and recognition. Offer training and development opportunities to enhance staff skills and self-efficacy; use supportive resources to stimulate motivation, meet psychological needs and strengthen coping capacity.
Dual-pathway (combined) interventions	Comprehensive initiatives that both reduce demands and increase resources, e.g., job crafting programmes (encouraging staff to proactively adjust their work to optimise demands and resource allocation). Engaging leadership (leadership behaviors that simultaneously reduce hindrance stressors while providing more supportive resources). Organisation-level actions to reduce work overload combined with individual-level programmes that build coping capacity.

### 4.2 Limitations of this study

If included studies differ substantially in intervention content and intensity, study design and methodological quality, follow-up duration, and outcome measures, such differences may undermine the stability of pooled effects and limit the generalisability of conclusions regarding the overall effectiveness of interventions for GP burnout. Although we will address heterogeneity to some extent through prespecified subgroup and sensitivity analyses, uncontrolled between-study differences and residual confounding may persist; therefore, subgroup findings should be interpreted with caution.

## Patient and public involvement

Patients and/or the public were not involved in the design, conduct, reporting or dissemination plans of this research.
