# Supplementary material for: Interventions to reduce occupational burnout in general practitioners: a systematic review and meta-analysis protocol
Source: Front Public Health. 2026 Mar 31;14:1748369. doi: 10.3389/fpubh.2026.1748369 (PMC13076574; doi:10.3389/fpubh.2026.1748369)
Supplement: Supplementary file 1 [file Supplementary_file_1.DOCX]

# **Appendix: Search Strategy**

| Databases | Search Strategy |
| --- | --- |
| PubMed | (("General Practitioners"[Mesh] OR "Family Practice"[Mesh] OR "Primary Health Care"[Mesh] OR general practitioner*[tiab] OR family physician*[tiab] OR family doctor*[tiab] OR primary care physician*[tiab] OR primary care provider*[tiab] OR primary care doctor*[tiab]) AND ("Burnout, Professional"[Mesh] OR "Occupational Stress"[Mesh] OR burnout[tiab] OR professional burnout[tiab] OR emotional exhaustion[tiab] OR job burnout[tiab] OR occupational stress[tiab] OR depersonalization[tiab]) AND (intervention*[tiab] OR prevention[tiab] OR preventive[tiab] OR program*[tiab] OR strategy[tiab] OR strategies[tiab] OR management[tiab] OR treatment[tiab] OR support[tiab] OR approach*[tiab])) |
| Web of Science | TS=(("general practitioner*" OR "family physician*" OR "family doctor*" OR "primary care physician*" OR "primary care provider*" OR "primary care doctor*") AND (burnout OR professional burnout OR emotional exhaustion OR job burnout OR occupational stress OR depersonalization) AND (intervention* OR prevention OR preventive OR program* OR strategy OR strategies OR management OR treatment OR support OR approach*)) |
| Embase | ('general practitioner'/exp OR 'family physician'/exp OR 'primary health care'/exp OR general practitioner*:ti,ab,kw OR family physician*:ti,ab,kw OR family doctor*:ti,ab,kw OR primary care physician*:ti,ab,kw OR primary care provider*:ti,ab,kw OR primary care doctor*:ti,ab,kw) AND ('burnout'/exp OR 'occupational stress'/exp OR burnout:ti,ab,kw OR professional burnout:ti,ab,kw OR emotional exhaustion:ti,ab,kw OR job burnout:ti,ab,kw OR depersonalization:ti,ab,kw) AND (intervention*:ti,ab,kw OR prevention:ti,ab,kw OR preventive:ti,ab,kw OR program*:ti,ab,kw OR strategy:ti,ab,kw OR strategies:ti,ab,kw OR management:ti,ab,kw OR treatment:ti,ab,kw OR support:ti,ab,kw OR approach*:ti,ab,kw) |
| CNKI | SU=("全科医生" OR "家庭医生" OR "社区医生" OR "基层医生" OR "初级保健医生" OR "初级保健") AND SU=("职业倦怠" OR "职业枯竭" OR "情绪耗竭" OR "职业压力" OR "去人格化") AND SU=("干预" OR "预防" OR "管理" OR "治疗" OR "支持" OR "方案" OR "策略" OR "措施" OR "项目" OR "应对") |
| Wanfang Data | 主题=("全科医生" OR "家庭医生" OR "社区医生" OR "基层医生" OR "初级保健医生" OR "初级保健") AND 主题=("职业倦怠" OR "职业枯竭" OR "情绪耗竭" OR "职业压力" OR "去人格化") AND 主题=("干预" OR "预防" OR "管理" OR "治疗" OR "支持" OR "方案" OR "策略" OR "措施" OR "项目" OR "应对") |
| VIP (Weipu) | ((M=("全科医生" OR "家庭医生" OR "社区医生" OR "基层医生" OR "初级保健医生" OR "初级保健") OR R=("全科医生" OR "家庭医生" OR "社区医生" OR "基层医生" OR "初级保健医生" OR "初级保健")) AND (M=("职业倦怠" OR "职业枯竭" OR "情绪耗竭" OR "职业压力" OR "去人格化") OR R=("职业倦怠" OR "职业枯竭" OR "情绪耗竭" OR "职业压力" OR "去人格化")) AND (M=("干预" OR "预防" OR "管理" OR "治疗" OR "支持" OR "方案" OR "策略" OR "措施" OR "项目" OR "应对") OR R=("干预" OR "预防" OR "管理" OR "治疗" OR "支持" OR "方案" OR "策略" OR "措施" OR "项目" OR "应对"))) |
